# Supplementary material for: Tristaenone A: A New Anti-Inflammatory Compound Isolated from the Australian Indigenous Plant Tristaniopsis laurina
Source: Molecules. 2022 Oct 5;27(19):6592. doi: 10.3390/molecules27196592 (PMC9571334; doi:10.3390/molecules27196592)
Supplement: Supplementary file 1 [file molecules-27-06592-s001.zip › molecules-1934210-supplementary.pdf]

Supporting Information

Tristaenone A: A new anti-inflammatory compound isolated from the Australian Indigenous plant *Tristaniopsis laurina*

Shintu Mathew,[†,‡] Xian Zhou,[†] Gerald Münch[†,‡] Francis Bodkin, [‡] Matthew J. Wallis, [§] Feng Li, [§] Ritesh Raju,[*,‡]

[†] NICM Health Research Institute, Western Sydney University, Penrith, NSW, Australia.

[‡] Department of Pharmacology, Western Sydney University, Campbelltown Campus, Sydney, Australia

[§] School of Science, Western Sydney University, Penrith, Sydney, NSW, Australia

Table of Contents

Figure S1. ^{1}H NMR spectrum of compound **1** (CD_3OD, 600 MHz) ……………………………………………4

Figure S2. ^{13}C NMR spectrum of compound **1** (CD_3OD, 600 MHz) ……………………………………………5

Figure S3. COSY spectrum of compound **1** (CD_3OD, 600 MHz) ……………………………………………6

Figure S4. HSQC spectrum of compound **1** (CD_3OD, 600 MHz) ……………………………………………7

Figure S5. HMBC spectrum of compound **1** (CD_3OD, 600 MHz) ……………………………………………8

Figure S6. UV-Vis spectrum for compound **1**……………………………………………9

Table S1. Crystallographic data for compound **1**…………………………………………… 10

Table S2. Fractional Atomic Coordinates ($\times 10^4$) and Equivalent Isotropic Displacement Parameters ($Å^2 \times 10^3$) for compound **1**…………………… 11

Figure S7. ^{1}H NMR spectrum of compound **2** (DMSO-d_6, 600 MHz) 13

Figure S8. ^{1}H NMR spectrum of compound **3** ($CDCl_3$, 600 MHz) 14

Figure S9. HRMS of compound **1** 15

Figure S10. HRMS of compound **2** 16

Figure S11. HRMS of compound **3** 17

Table S3. Downregulation of LPS and IFN- γ induced production of pro-inflammatory markers (NO and TNF- α) and cell viability of sequential extracts 18

Figure S1. ^{1}H NMR spectrum of compound **1** (CD_3OD, 600 MHz)

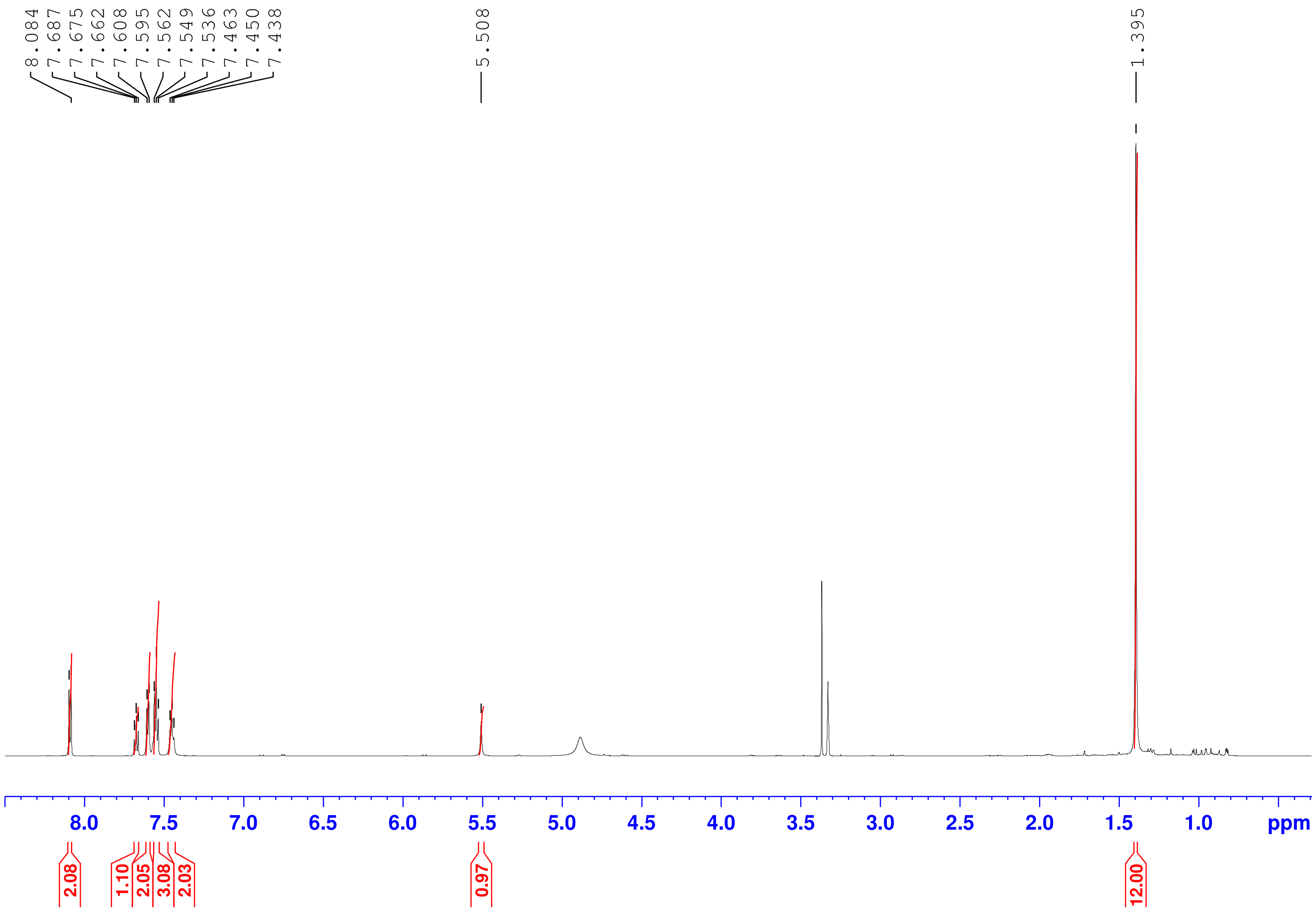

Figure S2. ^{13}C NMR spectrum of compound **1** (CD_3OD, 600 MHz)

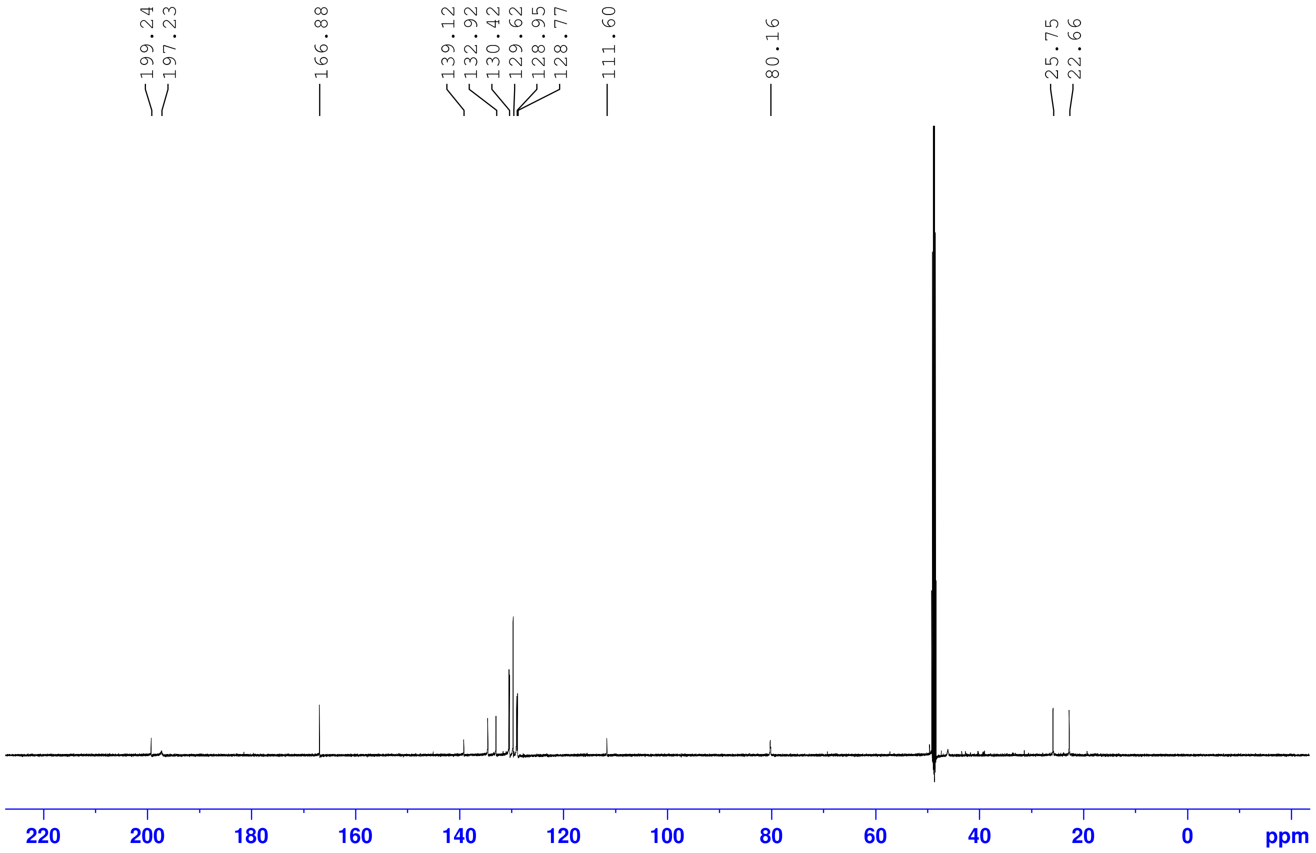

Figure S3. COSY spectrum of compound **1** (CD_3OD, 600 MHz)

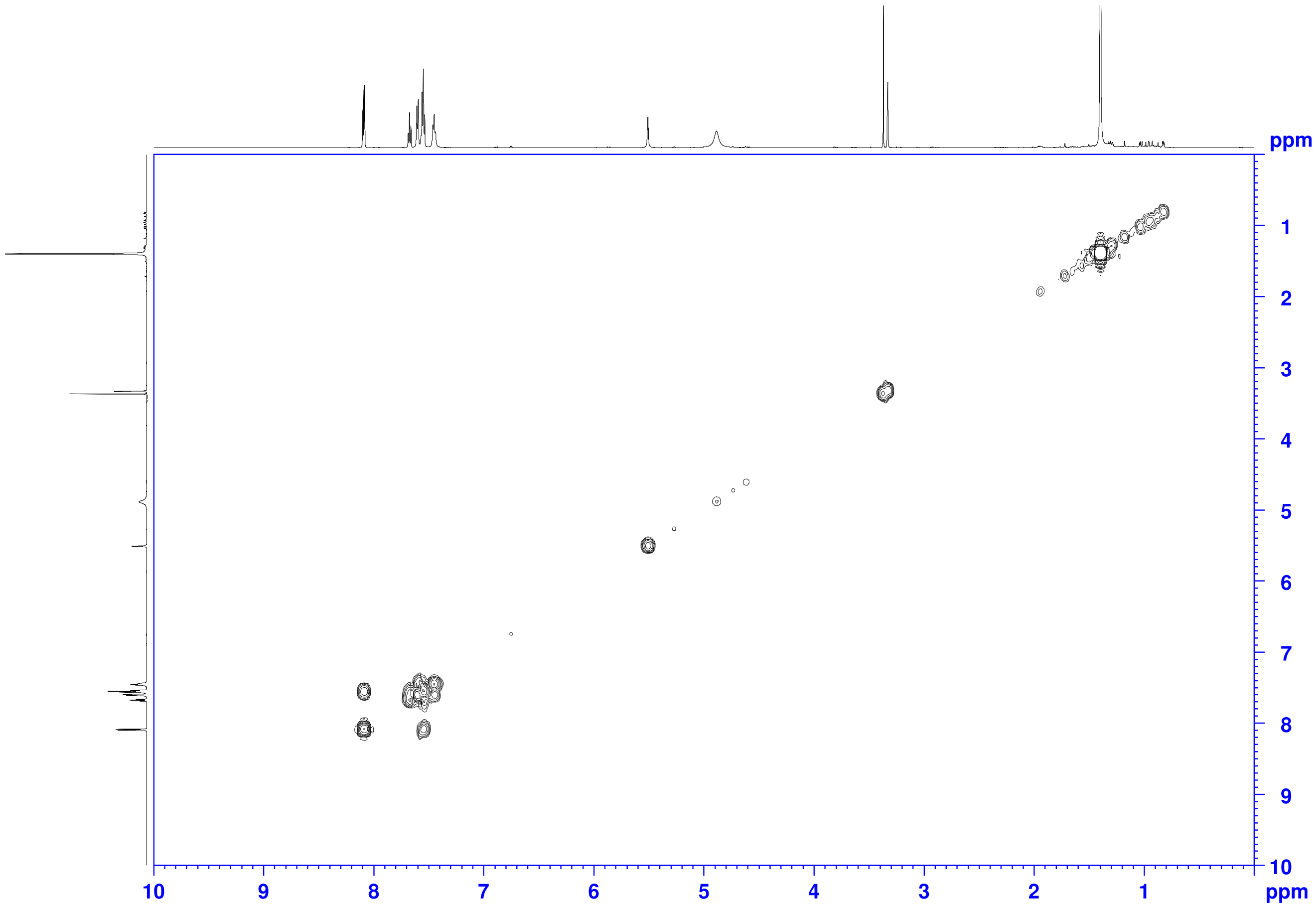

Figure S4. HSQC spectrum of compound **1** (CD_3OD, 600 MHz)

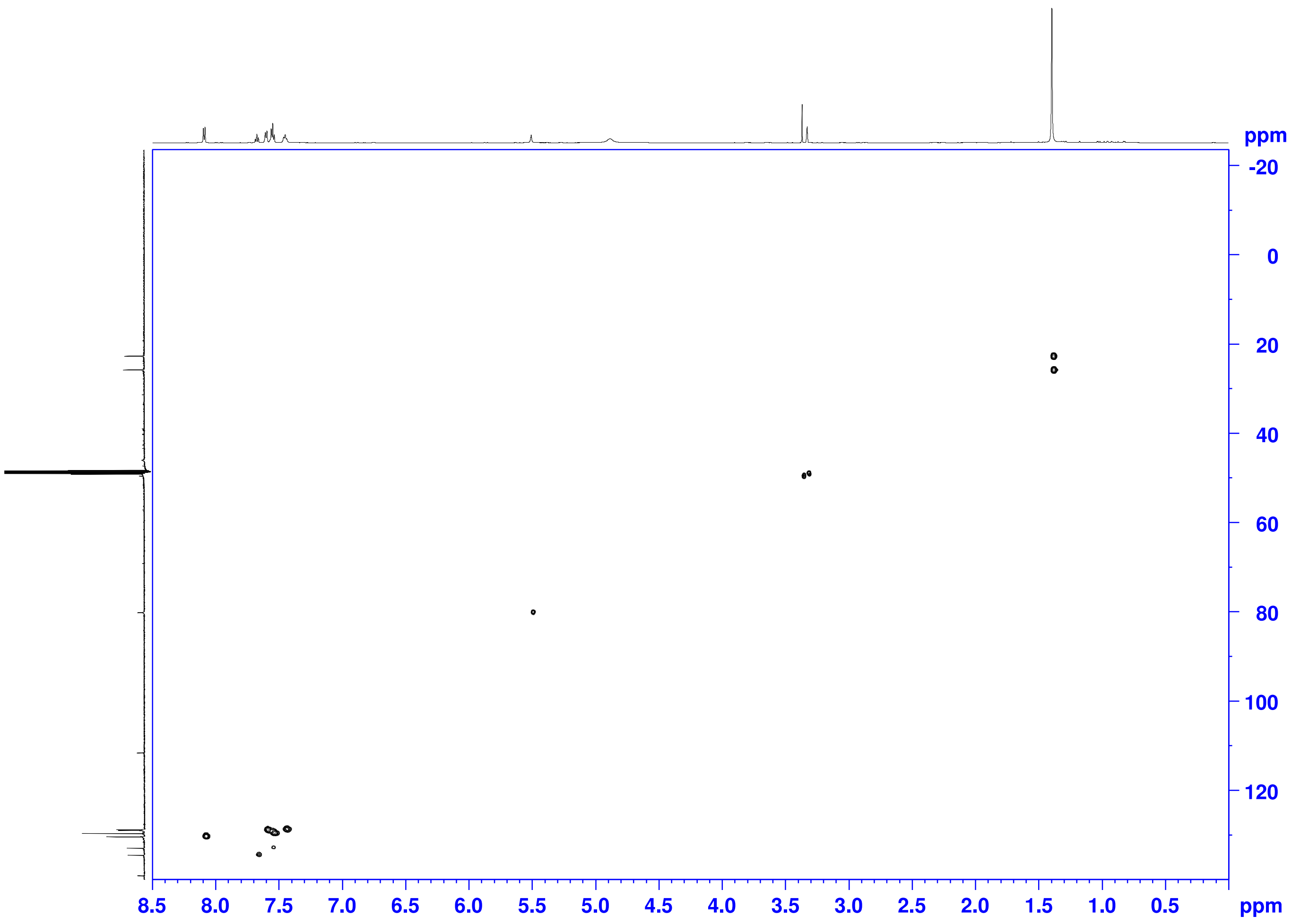

Figure S5. HMBC spectrum of compound **1** (CD_3OD, 600 MHz)

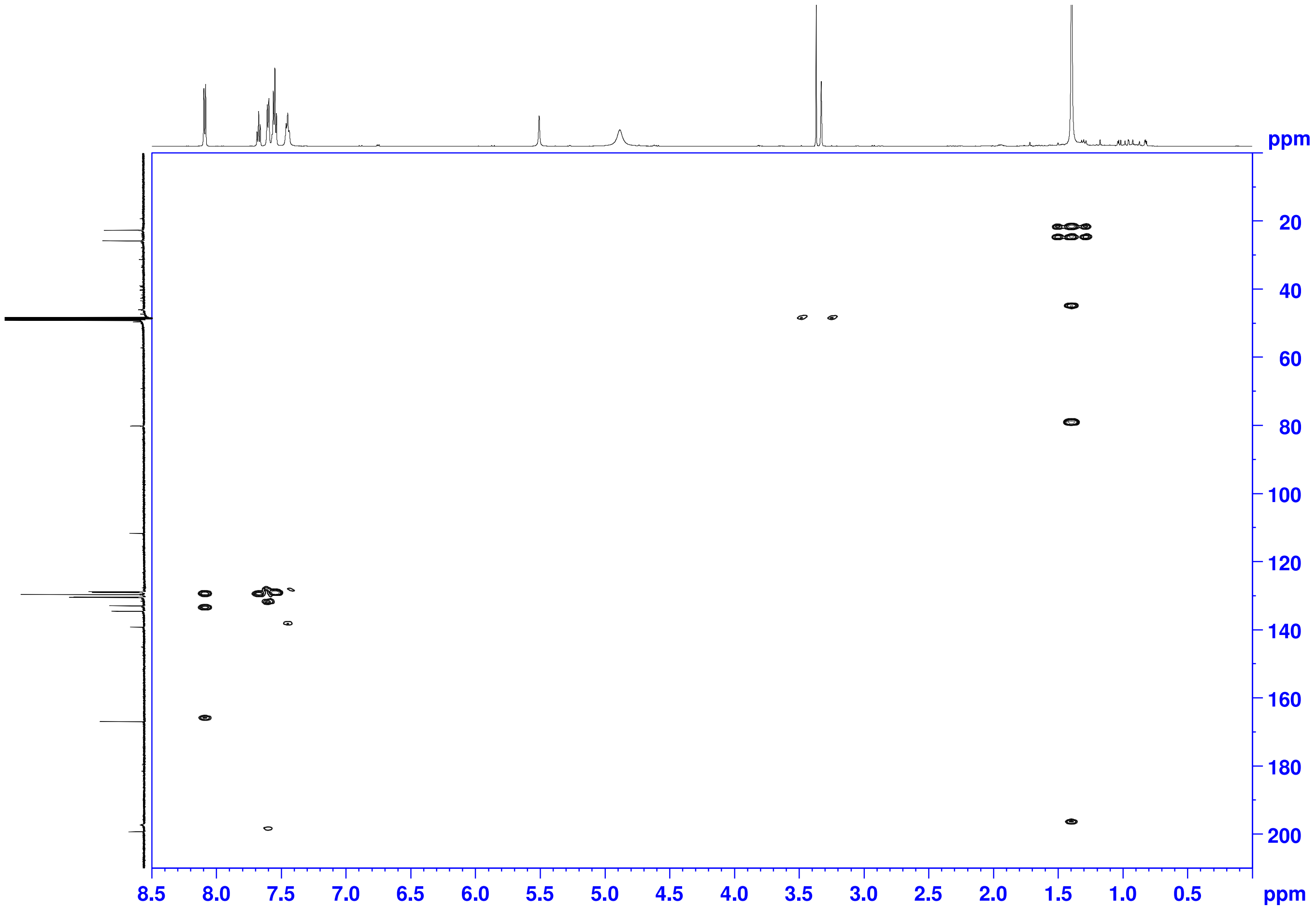

Figure S6. UV-Vis spectrum for compound **1**

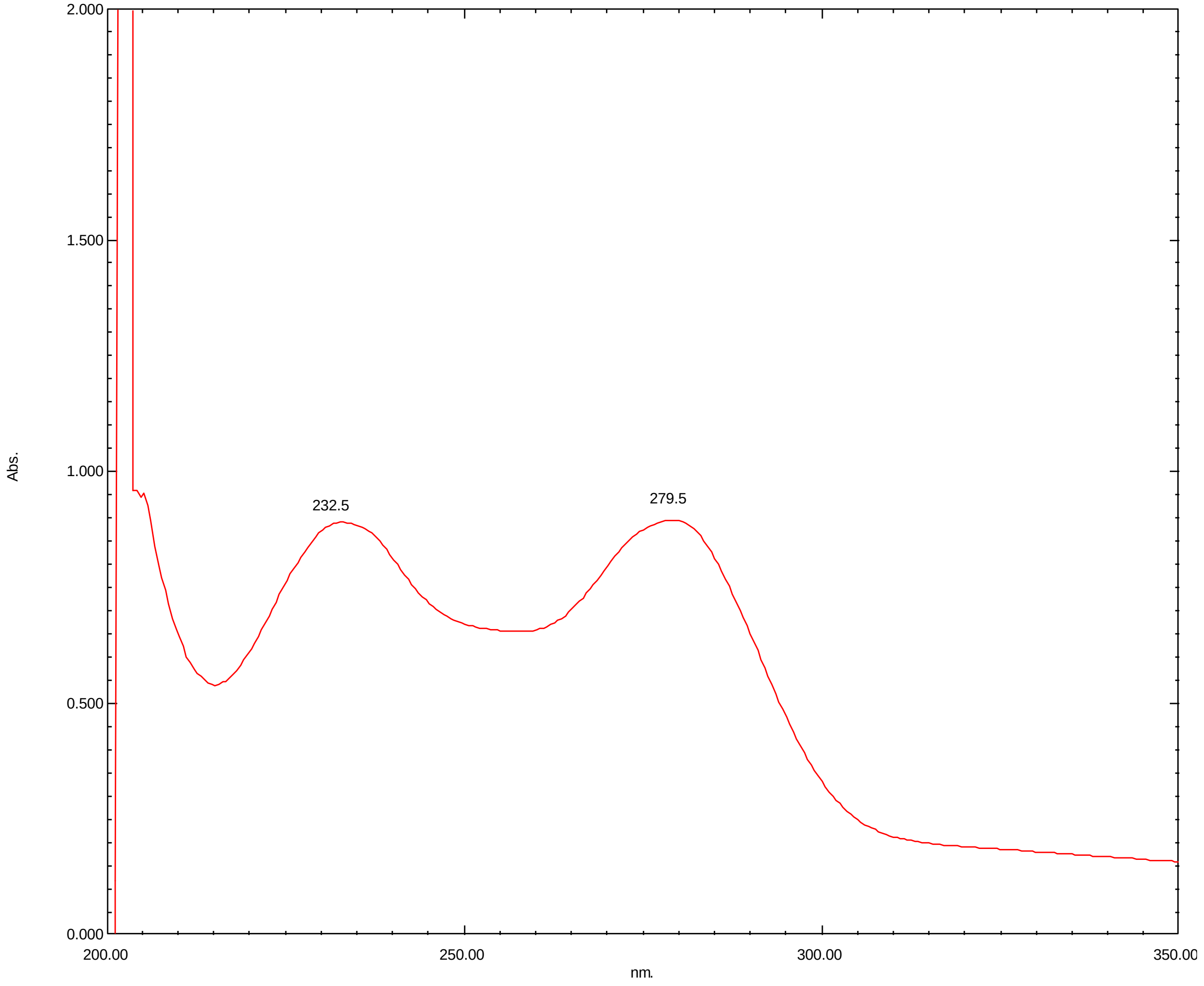

Table S1. Crystallographic data for compound **1**

CCDC number	2172244
Empirical formula	$C_{24}H_{24}O_5$
Formula weight	392.43
Temperature/K	100
Crystal system	monoclinic
Space group	$P2_1$
a/Å	6.0990(12)
b/Å	17.770(4)
c/Å	9.3760(19)
α/°	90
β/°	102.78(3)
γ/°	90
Volume/Å^3	991.0(4)
Z	2
ρ_{calc}g/cm^3	1.315
μ/mm^{-1}	0.092
F(000)	416.0
Crystal size/mm^3	0.1 × 0.1 × 0.01
Radiation	MoKα (λ = 0.71073)
2Θ range for data collection/°	4.454 to 57.272
Index ranges	$-7 \leq h \leq 8$, $-23 \leq k \leq 23$, $-11 \leq l \leq 11$
Reflections collected	12424
Independent reflections	3855 [R_{int} = 0.0453, R_{sigma} = 0.0321]
Data/restraints/parameters	3855/1/267

Goodness-of-fit on F^2	1.066
Final R indexes [I>=2σ (I)]	R_1 = 0.0323, wR_2 = 0.0825
Final R indexes [all data]	R_1 = 0.0329, wR_2 = 0.0833
Largest diff. peak/hole / e $Å^{-3}$	0.13/-0.24
Flack parameter	-0.1(3)

Table S2. Fractional Atomic Coordinates (×10^4) and Equivalent Isotropic Displacement Parameters ($Å^2$×10^3) for compound **1**. U_{eq} is defined as 1/3 of the trace of the orthogonalised U_{IJ} tensor.

Atom	*x*	*y*	*z*	U(eq)
O2	7487(2)	5551.3(8)	8672.2(14)	23.6(3)
O3	4659(3)	3392.4(8)	5800.1(15)	29.9(3)
O4	6615(3)	5522.0(8)	3502.6(16)	28.7(3)
O1	4381(2)	6120.7(9)	9115.3(16)	31.2(3)
O5	4330(3)	4565.5(9)	2008.8(15)	30.9(3)
C6	7943(3)	6330.4(11)	10736(2)	22.4(4)
C7	6378(3)	5998.0(11)	9448(2)	23.8(4)
C12	5177(3)	4059.2(12)	5890(2)	23.6(4)
C18	3822(4)	4277.5(12)	3116(2)	24.7(4)
C19	1955(4)	3723.6(12)	2849(2)	25.8(4)
C20	1635(4)	3277.3(12)	1587(2)	28.0(4)
C14	6202(3)	5199.8(12)	4667(2)	23.4(4)
C5	7139(4)	6909.4(11)	11478(2)	24.5(4)
C15	7146(3)	5620.1(12)	6064(2)	23.2(4)
C2	11580(3)	6441.4(13)	12352(2)	28.0(4)
C1	10176(3)	6092.3(11)	11173(2)	24.2(4)
C24	397(4)	3681.3(12)	3735(2)	27.6(4)

C3	10776(4)	7023.6(12)	13085(2)	28.5(4)
C4	8560(4)	7257.7(12)	12662(2)	27.0(4)
C9	6312(3)	4386.2(11)	7390(2)	22.9(4)
C13	4971(3)	4534.2(11)	4581(2)	22.9(4)
C8	6232(3)	5248.1(12)	7296(2)	22.4(4)
C21	-159(4)	2787.0(12)	1256(2)	31.7(5)
C10	8688(3)	4050.1(12)	7802(2)	26.7(4)
C22	-1696(4)	2743.0(12)	2148(2)	30.9(5)
C17	6239(4)	6436.1(12)	5874(2)	29.8(4)
C11	5015(4)	4133.0(13)	8540(2)	28.9(4)
C23	-1419(4)	3198.0(13)	3380(2)	29.6(4)
C16	9732(3)	5651.4(14)	6314(2)	30.6(5)

Figure S7. ^{1}H NMR spectrum of compound **2** (DMSO-d_6, 600 MHz)

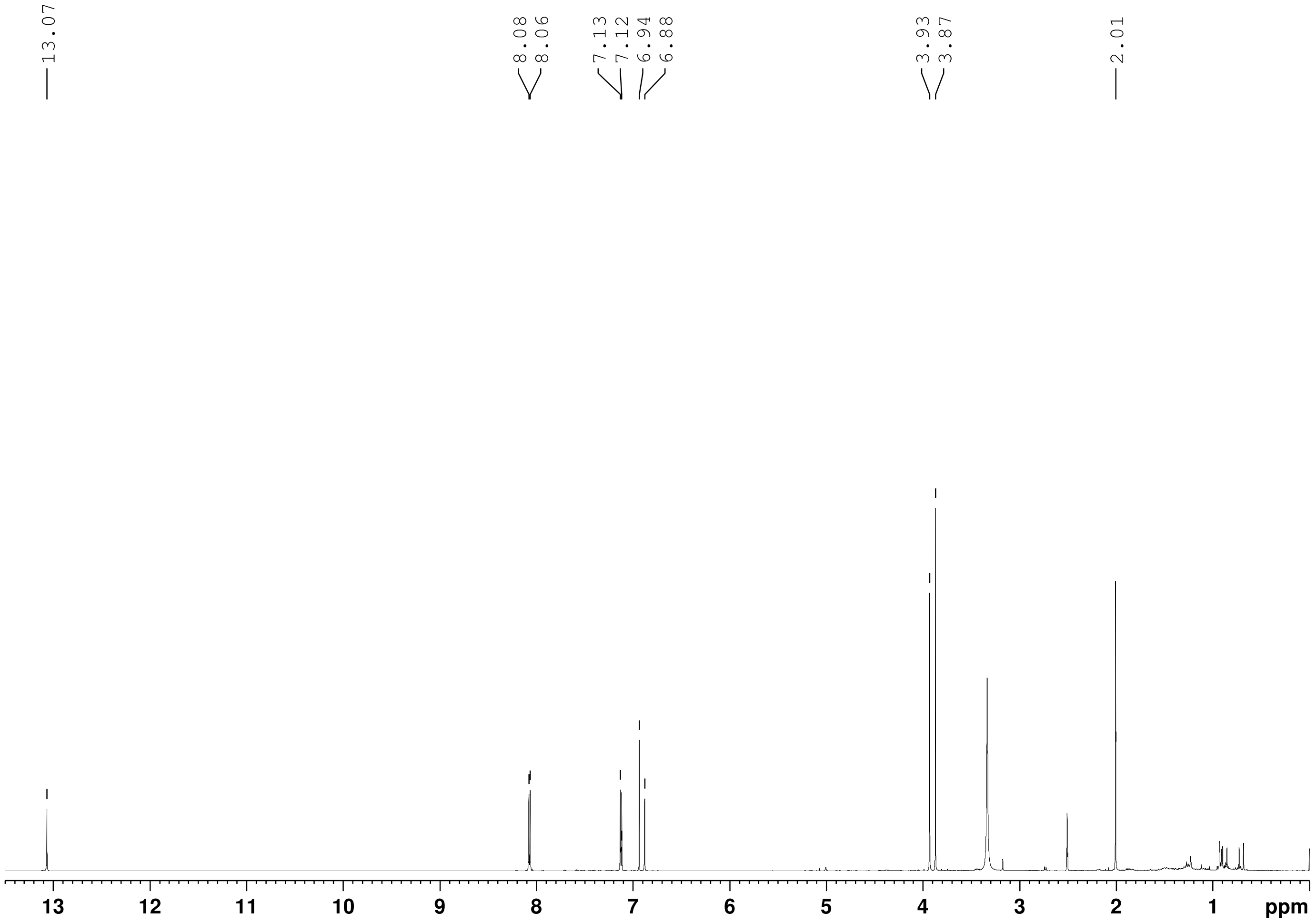

Figure S8. ^{1}H NMR spectrum of compound **3** ($CDCl_3$, 600 MHz)

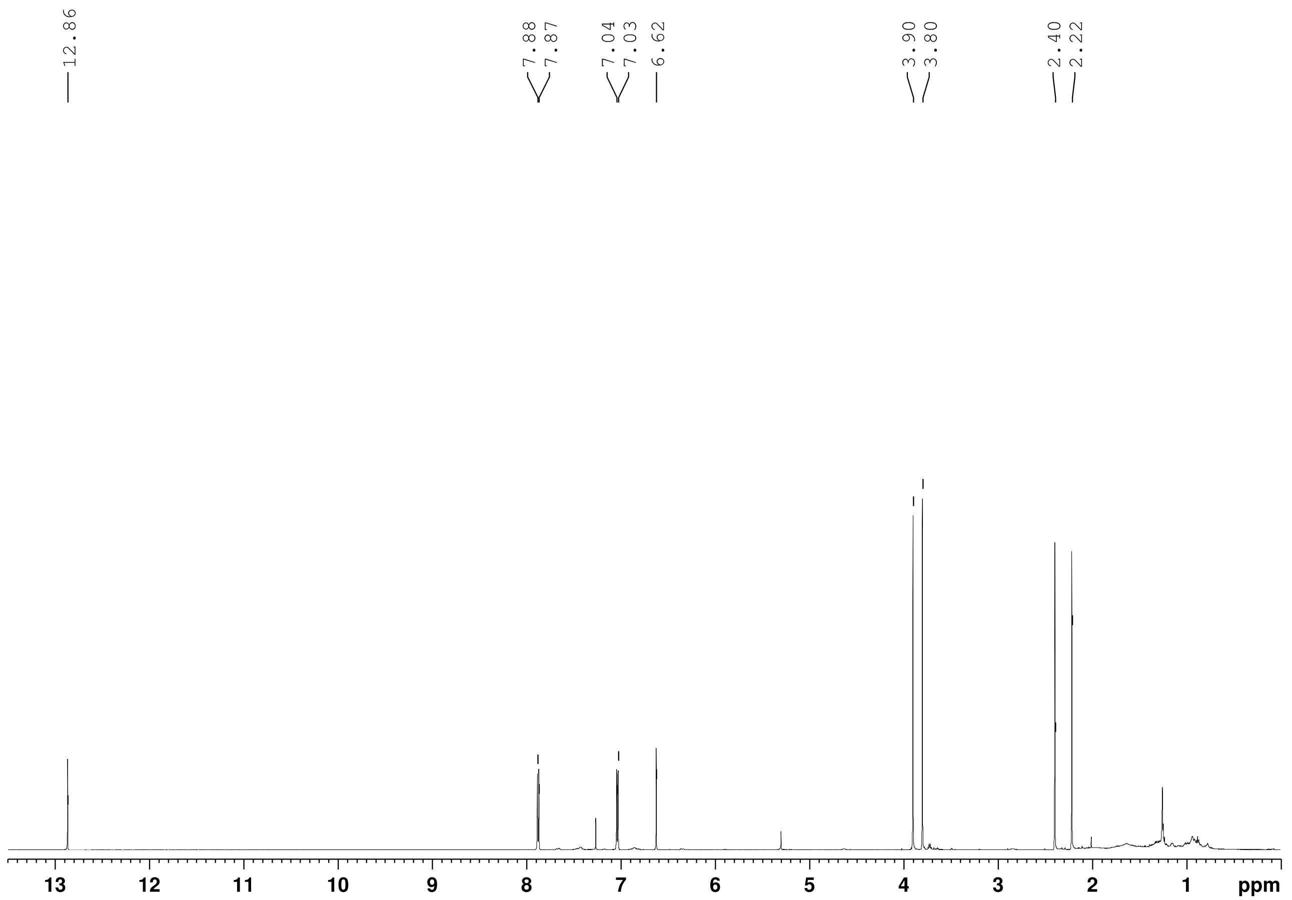

Figure S9. HRMS of compound **1**

Elemental Composition Report

Single Mass Analysis

Tolerance = 10.0 PPM / DBE: min = -1.5, max = 50.0
Element prediction: Off
Number of isotope peaks used for i-FIT = 3

Monoisotopic Mass, Even Electron Ions
178 formula(e) evaluated with 3 results within limits (all results (up to 1000) for each mass)
Elements Used:
C: 0-50 H: 0-130 O: 0-40 Na: 0-1
220301_TL_F-14A 81 (1.604) Cm (26:82)
1: TOF MS ES+

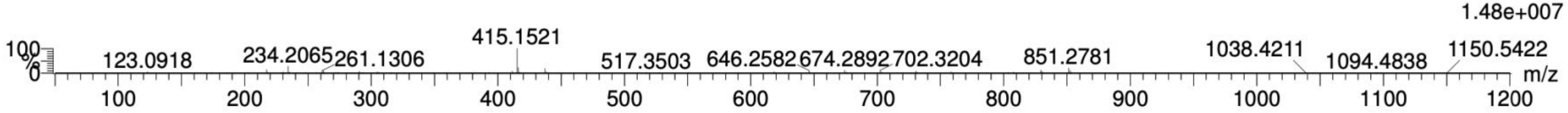

Minimum:				-1.5
Maximum:		10.0	10.0	50.0

Mass	Calc. Mass	mDa	PPM	DBE	i-FIT	Norm	Conf(%)	Formula
415.1521	415.1521	0.0	0.0	12.5	2094.6	0.056	94.60	C24 H24 O5 Na
	415.1545	-2.4	-5.8	15.5	2097.5	2.958	5.19	C26 H23 O5
	415.1487	3.4	8.2	24.5	2100.8	6.178	0.21	C33 H19

Figure S10. HRMS spectrum for compound **2**

Elemental Composition Report

Page 1

Single Mass Analysis

Tolerance = 100.0 PPM / DBE: min = -1.5, max = 50.0
Element prediction: Off
Number of isotope peaks used for i-FIT = 3

Monoisotopic Mass, Even Electron Ions
124 formula(e) evaluated with 18 results within limits (all results (up to 1000) for each mass)
Elements Used:
C: 0-50 H: 0-100 O: 0-30 Na: 0-1
220301_TL_F-13A 80 (1.567) Cm (80)
1: TOF MS ES+

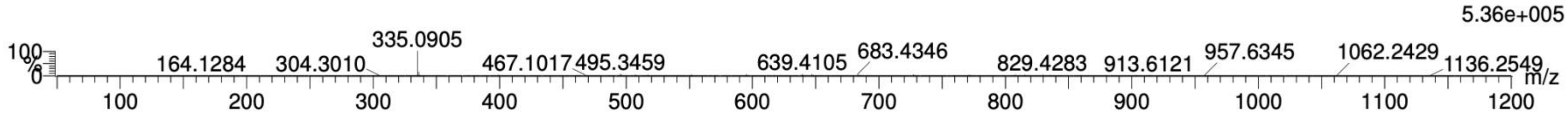

		mDa	PPM	DBE				
Minimum:				-1.5				
Maximum:		10.0	100.0	50.0				

Mass	Calc. Mass	mDa	PPM	DBE	i-FIT	Norm	Conf(%)	Formula
335.0905	335.0895	1.0	3.0	10.5	682.4	4.588	1.02	C18 H16 O5 Na

Figure S11. HRMS spectrum for compound **3**

Elemental Composition Report

Single Mass Analysis

Tolerance = 100.0 PPM / DBE: min = -1.5, max = 50.0
Element prediction: Off
Number of isotope peaks used for i-FIT = 3

Monoisotopic Mass, Even Electron Ions
43 formula(e) evaluated with 1 results within limits (all results (up to 1000) for each mass)
Elements Used:
C: 19-19 H: 0-100 O: 0-30 Na: 0-1
220315_TL-16A 65 (1.294) Cm (65:67)
1: TOF MS ES+

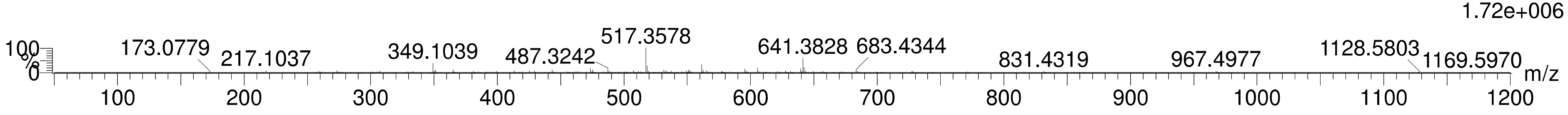

Minimum:				-1.5				
Maximum:		10.0	100.0	50.0				
Mass	Calc. Mass	mDa	PPM	DBE	i-FIT	Norm	Conf(%)	Formula
349.1039	349.1052	-1.3	-3.7	10.5	1135.3	n/a	n/a	C19 H18 O5 Na

Table S3. Downregulation of LPS and IFN- γ induced production of pro-inflammatory markers (NO and TNF- α) and cell viability of sequential extracts

**Sequential Extracts**	**Inhibition of nitric oxide production (μg/mL)**	**Cell viability (μg/mL)**
Hexane	3.11±0.45	12.30±2.99
DCM	2.68±0.48	7.90±0.27
EtOAc	4.08±0.39	17.93±2.16
MeOH	30.88±4.06	>100
Water	29.36±4.88	>100
